# Combined nerve and vascular ultrasound in thoracic outlet syndrome: A sensitive method in identifying the site of neurovascular compression

**DOI:** 10.1371/journal.pone.0268842

**Published:** 2022-05-25

**Authors:** Peter Dollinger, Josef Böhm, Zsuzsanna Arányi

**Affiliations:** 1 Dept. of Vascular Surgery, DRK Kliniken Berlin Mitte, Berlin, Germany; 2 Neurologische Praxis, Dr Josef Böhm, Berlin, Germany; 3 Dept. of Neurology, Semmelweis University, Budapest, Hungary; University Hospital-Eppendorf, GERMANY

## Abstract

We investigated the diagnostic utility of combined nerve and vascular ultrasound in thoracic outlet syndrome (TOS) in a retrospective cohort study on two sites, involving 167 consecutive patients with the clinical symptoms suggestive of neurogenic and/or vascular TOS, and an age- and sex-matched control group. All patients and control subjects underwent nerve ultrasound of the supraclavicular brachial plexus to look for fibromuscular anomalies / compression of the brachial plexus in the scalenic region, and vascular ultrasound of the infraclavicular subclavian artery with the arm in neutral and abducted position, serving as an indicator for costoclavicular compression of the neurovascular bundle. Based on clinical symptoms, neurogenic TOS (81%) was the most frequent *type* of TOS, followed by combined neurogenic and arterial TOS (8%). The frequency of abnormal nerve and/or vascular ultrasound findings differed significantly from the control group (P<0.00001). The pooled sensitivity was 48% for nerve ultrasound, 85% for vascular ultrasound, and 94% when combined. Among the findings, the fibromuscular ‘wedge-sickle sign’, indicating compression of the lower trunk in the scalenic region by a congenital fibromuscular anomaly (e.g. Roos ligaments), showed the highest specificity (100%). A bony ‘wedge-sickle sign’ was also delineated, where lower trunk compression is caused by the neck of the 1^st^ rib. As implied by the higher sensitivity of vascular ultrasound, the most common *site* of compression was the costoclavicular space, but multilevel compression was also frequently observed. In summary, combined nerve and vascular ultrasound is a sensitive and reliable method to support the diagnosis of TOS. It can also identify the site(s) of compression, with obvious therapeutic consequences.

## Introduction

Thoracic outlet syndrome (TOS) is an umbrella term for a group of disorders characterized by the compression of the neurovascular bundle of the upper limb at various levels of the thoracic outlet region, including the supraclavicular scalene triangle, the costoclavicular space, and the retropectoral space [1]. The diagnosis of TOS is usually straightforward when its three major manifestations, neurogenic, arterial and venous TOS present in their extreme forms, namely with wasting of small hand muscles (Gilliatt-Sumner hand), subclavian artery aneurysm with distal embolism, and thrombosis of the subclavian vein (Paget-Schrötter syndrome), respectively [2–4]. On the other hand, less severe forms are associated with a range of intermittent and / or subjective symptoms as a result of brachial plexus irritation, position dependent vascular compromise of the arm, or a combination of both [5]. These symptoms may pose differential diagnostic problems, which contributes to the many controversies TOS is historically surrounded with, regarding its prevalence, diagnosis, clinical boundaries, and treatment. Wilbourne [6] has even introduced the concept of ‘nonspecific’ or ‘disputed’ TOS for cases with pain but without neurological deficit, as opposed to ‘true’ neurogenic TOS with clinical deficit. Improving the diagnostic accuracy of both the neurogenic and vascular components of TOS, including the determination of the exact site of compression, is one important step towards understanding the disorder in its entirety.

The diagnosis of TOS is a complex, interdisciplinary process, involving—among others–neurologists, radiologists and vascular specialists. Ultrasound has been long time applied for the assessment of blood vessels, and lately also for the assessment of nerves. Recently, we have described the ultrasonographic ‘wedge-sickle sign’ as an imaging proof for the compression of the lower trunk of the brachial plexus in the supraclavicular region causing neurogenic TOS [7]. The aim of the present retrospective study was to investigate the diagnostic utility and reliability of combined nerve ultrasound of the brachial plexus and Duplex ultrasound of the subclavian artery in patients with symptoms suggestive for neurogenic and/or vascular TOS.

## Materials and methods

Consecutive patients between 2017 and 2021 were included in the study carried out on two sites: Neurologische Privatpraxis–Dr Josef Böhm + Dept. of Vascular Surgery, DRK Kliniken Berlin Mitte in Berlin (Site A), and Dept. of Neurology, Semmelweis University in Budapest (Site B). Inclusion criteria of patients in the study were the clinical symptoms and signs suggestive of neurogenic [8] or vascular TOS [9], irrespective of symptom duration, and the exclusion of alternative diagnoses, such as carpal tunnel syndrome, ulnar nerve lesion, cervical radiculopathy or shoulder disease. Patients with previous surgery or major trauma in the region were excluded. All patients underwent nerve ultrasound of the brachial plexus and vascular ultrasound of the subclavian artery (described in detail below) as part of their routine clinical assessment. The subclavian vein was also assessed in most patients, but these results were not included in the analysis. In addition, on site A all patients underwent angiography as well, as part of their work-up towards surgery. Retrospective analysis was performed using fully anonymized patient data with the approval from the relevant Review Boards on both sites (Ethik-Kommission der Ärztekammer Berlin, Semmelweis University Regional and Institutional Committee of Science and Research Ethics).

### Ultrasonography

The scanning was performed by the authors. On Site A, a Mindray Resona 7 device with L20-5U and L11-3U MHz linear array transducers (Mindray, Nanshan, China) and GE Logiq E9 XDclear device with 9 L-D 2.5–9.5 MHz transducer (General Electric, Boston, USA), on Site B a Philips Epiq 5 device with L18-5 and L12-5 MHz linear array transducers (Philips, Amsterdam, The Netherlands) were used.

First, the whole supraclavicular portion of the brachial plexus was scanned on the symptomatic side(s) in supine or sitting position (depending on personal preference of the examiner), with the head turned in the opposite direction, according to standard methods and landmarks [10, 11]. Axial scanning was started at the supraclavicular fossa, where the lower trunk of the brachial plexus was identified immediately lateral to the subclavian artery. Scanning was continued cranially in the interscalenic region up to the foraminal level of the C5 root. Special attention was paid to the lower trunk of the brachial plexus, and any structures in its vicinity. Abnormalities, such as compression of the lower trunk (‘wedge-sickle sign’), and aberrant muscles (medial insertion of the middle scalene muscle on the 1^st^ rib, presence of scalenus minimus muscle between the lower trunk and the subclavian artery) were noted.

The subclavian artery was then scanned, with the patient in sitting position, in B-mode, colour Doppler and PW mode in the immediate infraclavicular region, as it leaves the costoclavicular space. Blood flow velocity was measured first with the arm in the neutral (adducted) position, then at 90° abduction and external rotation (AER). Hemodynamically significant costoclavicular impingement of the artery at the AER position was established if the blood flow velocity increased by at least two-fold (significant dynamic stenosis) [12], the blood flow stopped altogether (dynamic occlusion), or showed a poststenotic tardus-parvus pattern. As the compression on the artery is dynamic in nature and even slight positional adjustments may effect blood flow, the position of the arm was fine-tuned based on the provoked symptoms and blood flow changes, before the final measurement was made. Clinical symptoms provoked by the manoeuvre were noted, as well as any change in the shape of the artery.

An age and sex-matched control group of 50 individuals without any subjective or objective symptoms and signs suggestive of TOS, was compiled prospectively on both sites. Control subjects underwent ultrasound of the brachial plexus and the subclavian artery as described above on the right side.

### Angiography

On Site A, angiography of the subclavian artery was also carried out after ultrasonographic assessment in all patients as part of their work-up for TOS surgery. Via puncture of the common femoral artery and using 4 F sheath introducers, both subclavian arteries were catheterized selectively. The table was then turned to the upright position and serial X-ray images were captured while contrast medium was injected in 3 standard arm positions (0° arm hanging, 90° abducted/externally rotated and arm 180° elevated).

### Data analysis

To test data reliability, results from the two sites were analysed both separately and pooled. Descriptive statistics (mean, standard deviation, and range) were applied to describe the age at diagnosis (assessment) and the duration of symptoms. The frequencies of clinical symptoms and signs, neurogenic and vascular ultrasonographic abnormalities, as well as of angiographic abnormalities were determined. The sensitivity, specificity, positive and negative predictive values were calculated for the neurogenic and vascular ultrasonographic abnormalities, separately and combined, using clinical symptoms as a gold standard. Finally, the frequencies of the different compression sites and clinical categories of TOS were determined.

The two-tailed unpaired t-test was used to compare continuous variables. The chi-square statistic was used to test for association between categorical variables. Statistical significance was set at p< 0.05.

## Results

*Table 1* shows the summary of demographic and clinical data. The patient groups included 57 and 110 patients on sites A and B, respectively, 167 in total. Data show that a typical patient with TOS was a young female presenting with arm pain and numbness in the ulnar distribution indicating neurogenic TOS, but without neurological deficit. A minority of patients had earlier or ongoing thrombosis of the subclavian vein, but signs of arterial disease of the upper limb were not observed in our cohort.

**Table 1:** Summary of demographic and clinical data.

	Site A	Site B	Sites A+B	Control	P value
	(n = 57)	(n = 110)	(n = 167)	(n = 50)	
**Age at diagnosis, years**	40.9 ± 12.2	36.7 ± 13.4	**38.1 ± 13.1**	42.5 ± 14.4	0.053 (sites A v. B)
mean ± SD (range)	(16–63)	(14–76)	**(14–76)**	(12–78)	0.044 (A+B v. control)
**Sex, % of females**	74	81	**77**	76	
**Symptom duration, months**	37.8 ± 43.9	29.1 ± 37.3	**32.2 ± 39.8**	N/A	0.17 (sites A v. B)
mean ± SD (range)	(1–240)	(1–240)	**(1–240)**		
**Affected side, % of pts**	21/21/58	41/33/25	**35/29/36**	N/A	
R/L/B					
**Complaints, % of pts**				N/A	
• Ulnar numbness / pain	79	55	**63**		
• Shoulder-arm-scapular pain	77	63	**68**		
• Nocturnal symptoms	35	15	**22**		
• Coldness / pallor / exercise induced	21	7	**12**		
• Subclavian vein thrombosis	7	3	**6**		
**Neurological deficit, % of pts**				N/A	
• None	91	75	**81**		

pts: patients; SD: standard deviation; R/L/B: right/left/bilateral; N/A: not applicable

### Ultrasonography

**Fig 1:**
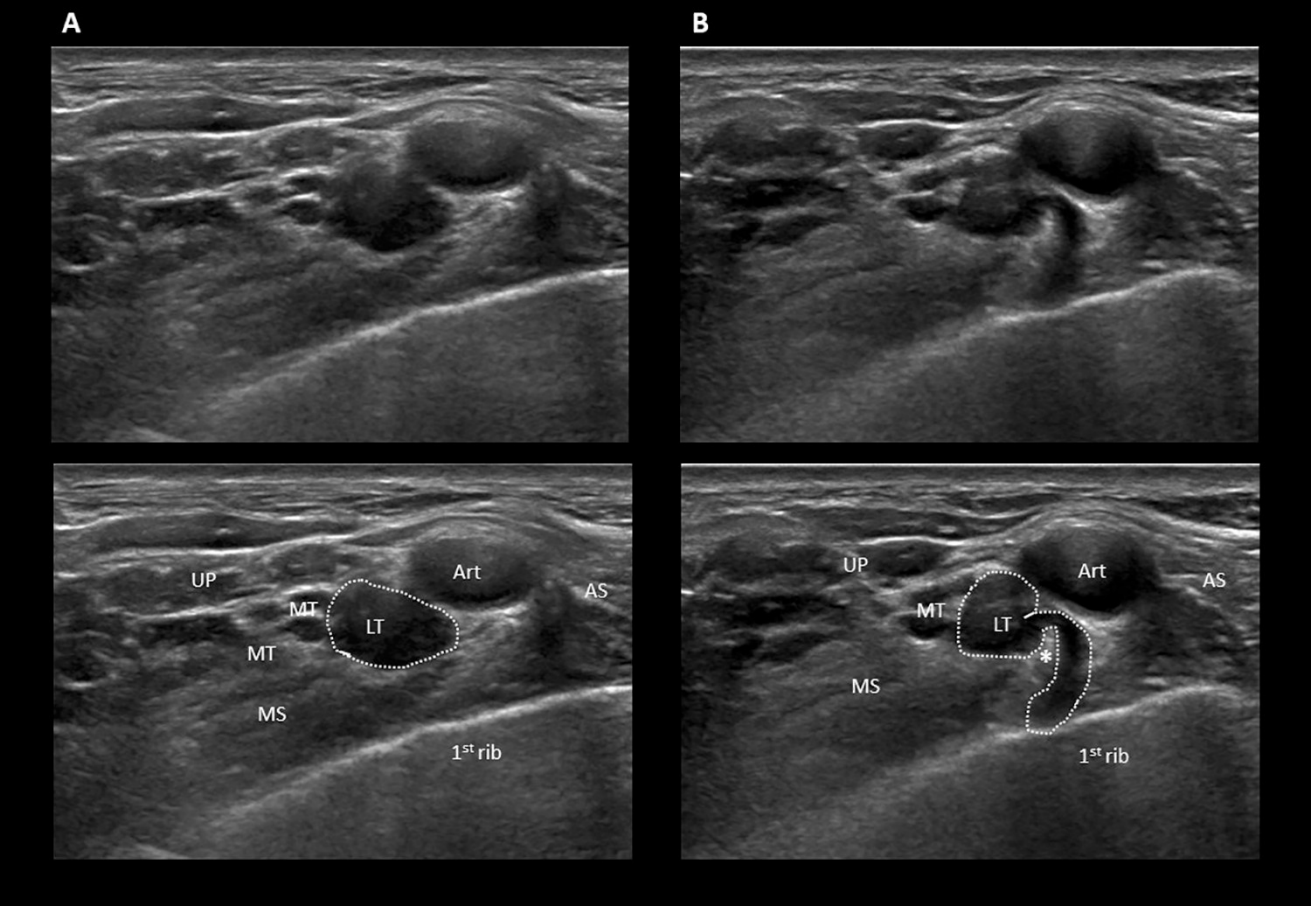
The fibromuscular ‘wedge-sickle sign’. Axial images showing the right brachial plexus in the supraclavicular fossa in patient with neurogenic (supraclavicular) TOS (see also S1 Video). Image **B** is cephalad relative to image **A**. In **B**, note the hyperechoic, ‘wedge’ shaped fibromuscular structure (asterisk) along the caudal medial edge of the middle scalene muscle, indenting (compressing) the lower trunk from the infero-lateral direction, which thus assumes the shape of a ‘sickle’ and becomes swollen and hypoechoic. The fibromuscular structure in this case is a fibrous band connecting the anterior tip of a rudimentary cervical rib (appearing further cephalad as seen in S1 Video) with the first rib (Type 1 Roos ligament). Caudal to the compression (**A**) the lower trunk is still swollen and hypoechoic, but its shape is round. The middle and upper trunks are normal. Note also the medial insertion of the middle scalene muscle on the 1^st^ rib, forming a V-shaped sling with the anterior scalene muscle and elevating the brachial plexus and the subclavian artery, further restricting space. LT: lower trunk; MD: middle trunk; UP: upper trunk; MS: middle scalene muscle; AS: anterior scalene muscle; Art: subclavian artery.

**Fig 2:**
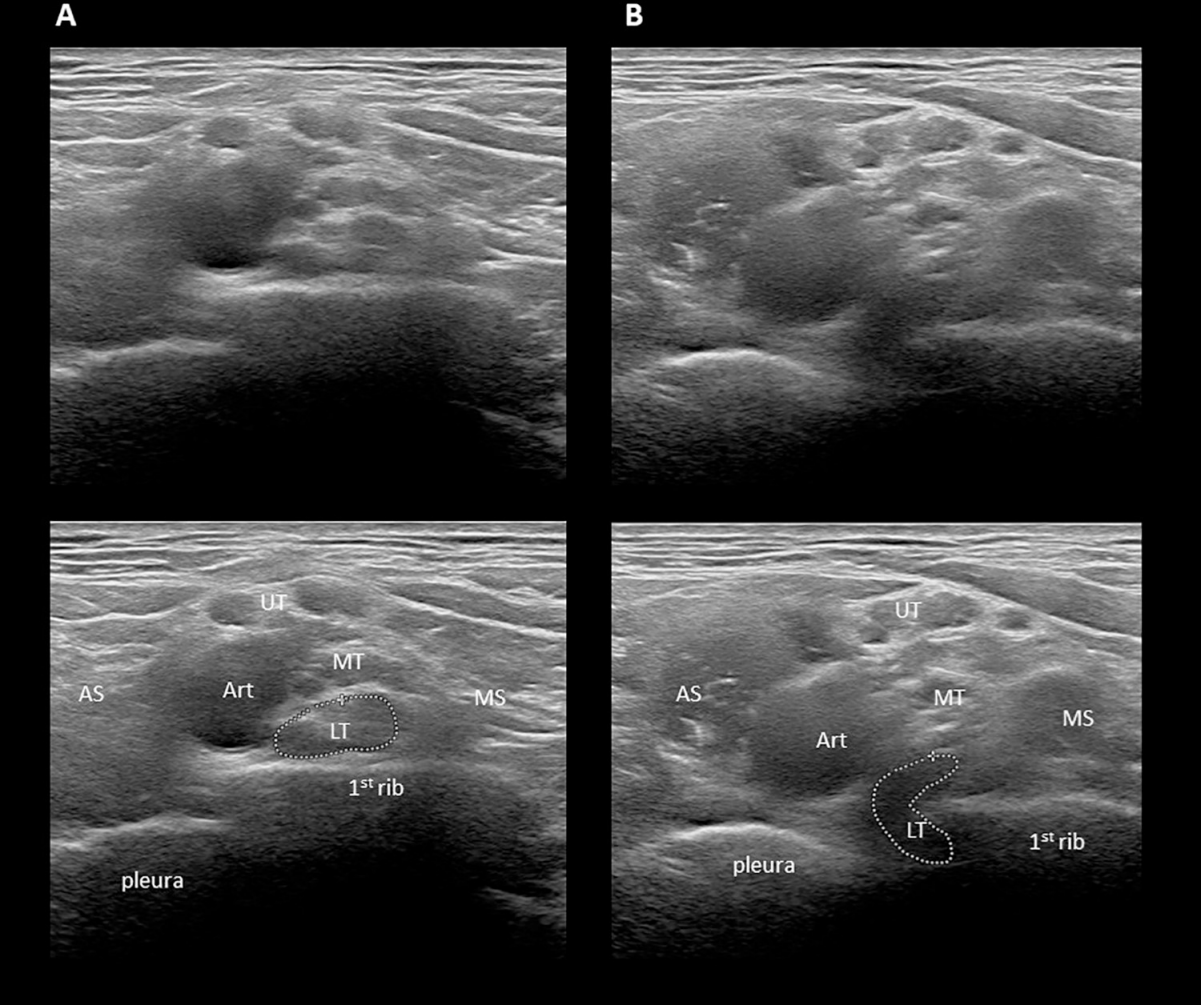
The bony ‘wedge-sickle sign’. Axial images showing the right brachial plexus in the supraclavicular fossa in patient with neurogenic (supra- + costoclavicular) TOS. Image **B** is cephalad relative to image **A**. In **A**, note the elevated position of the 1^st^ rib. In **B**, note that the medial edge of the 1^st^ rib indents the lower trunk from the lateral direction, which assumes a sickle shape and is moderately hypoechoic and swollen. LT: lower trunk; MD: middle trunk; UP: upper trunk; MS: middle scalene muscle; AS: anterior scalene muscle; Art: subclavian artery.

**Fig 3:**
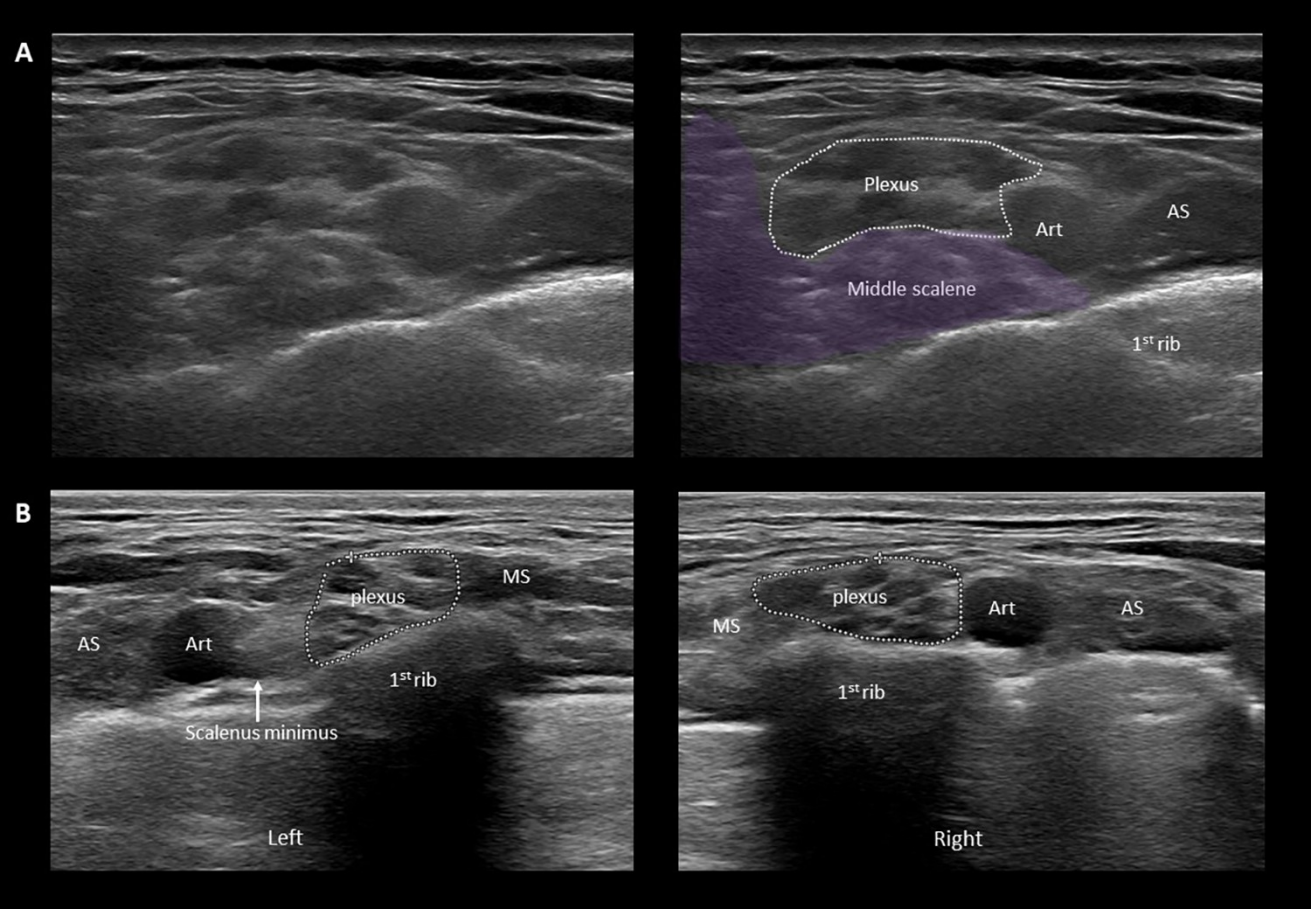
Muscle anomalies in the supraclavicular region. **A**. Axial images of the supraclavicular fossa showing the anomalous medial insertion of the middle scalene muscle on the 1^st^ rib in a patient with neurogenic + arterial TOS. Note how the muscle elevates the brachial plexus and has a space-occupying effect. Compare to image **A** of Fig 2. Further cephalad, a ‘wedge-sickle sign’ is also present (not shown). **B**. Axial images of the supraclavicular fossa showing an accessory scalenus minimus muscle in a patient with neurogenic + arterial (supra- + costoclavicular) TOS. Note that on the left side, a supernumerary muscle is present between the subclavian artery and the brachial plexus, which has a space-occupying effect. On the right side, the brachial plexus is located immediately adjacent to the artery. Note also the elevated position of the 1^st^ rib. MS: middle scalene muscle; AS: anterior scalene muscle; Art: subclavian artery.

**Fig 4:**
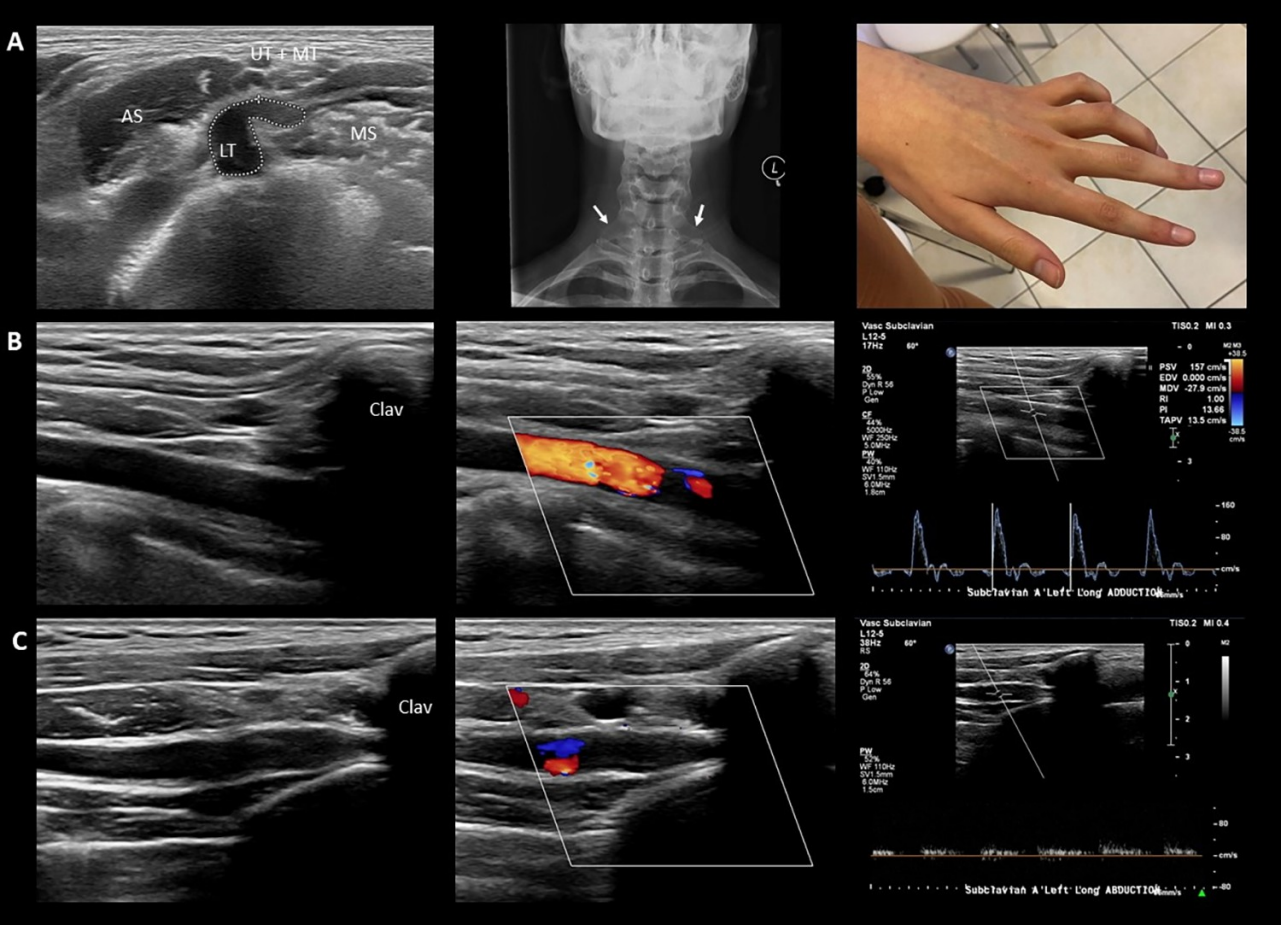
Multilevel compression in neurogenic TOS. Images of a 20-year-old female patient with neurogenic (supra- + costoclavicular) TOS, demonstrating both supraclavicular ‘wedge-sickle sign’ and costoclavicular impingement. Her complaints started 6 months earlier with nocturnal numbness of the left 4-5^th^ fingers, followed by the development of claw hand, indicating dysfunction of the 4-5^th^ lumbricalis / interosseous (lower trunk innervated) muscles. Apart from the claw hand and minor sensory disturbance in the distribution area of the lower trunk, she had no neurological deficit. Electrophysiological assessment was normal. **A**. The image on the left is an axial scan of the supraclavicular fossa showing a typical fibromuscular ‘wedge-sickle sign’. Note the sickle-shaped, swollen and hypoechoic lower trunk. The hyperechoic fibromuscular structure in this case is a fibrous band connecting an elongated transverse process of the 7^th^ cervical vertebra (arrows in the middle image) with the 1^st^ rib (Type 2 Roos ligament). The image on the right shows her ‘claw hand’. **B** and **C** show the vascular ultrasound (B mode, Colour Doppler and PW mode) of the left subclavian artery in the immediate infraclavicular region in neutral arm position (**B**) and during arm abduction (**C**). Note that during arm abduction the artery is narrowed, followed by a poststenotic dilation (‘funnel sign’) as it leaves the costoclavicular space, associated with almost complete occlusion. The patient’s complaints were exacerbated during the manoeuvre. LT: lower trunk; MD: middle trunk; UP: upper trunk; MS: middle scalene muscle; AS: anterior scalene muscle; Clav: clavicula.

*Table 2* shows the summary of all findings, and *Table 3* the sensitivity, specificity, positive and negative predictive values of the nerve, vascular and combined ultrasound findings. Results from the two sites did not differ significantly with respect to nerve and combined nerve and vascular ultrasound findings, but there was a marginally significant (p = 0.038125) difference with respect to vascular ultrasound findings, the sensitivity being higher on Site A. All results in the TOS group differed highly significantly from the control group. The sensitivity was the lowest for nerve ultrasound (48%), higher (85%) for vascular ultrasound, but the highest sensitivity was found (94%) when the two methods were combined. In 35% of patients, both nerve and vascular abnormalities were present. *Figs 1–4* and *S1 Video* show examples of nerve ultrasound findings. It is to be noted that although the fibromuscular ‘wedge-sickle sign’ (*Figs 1* and *4*, *S1 Video*) was of relatively low sensitivity (23%), it was the only finding never observed in the control group resulting in 100% specificity and positive predictive value for TOS. In 16/167 patients, nerve ultrasound findings were multiple (not shown in Table 2). Typically, the ‘wedge-sickle sign’ and the medial insertion of the middle scalene muscle occurred together (*Figs 1* and *3, S1 Video*).

**Table 2:** Summary of ultrasonographic and angiographic findings.

	Site A	Site B	Sites A+B	Control	P value
	(n = 57)	(n = 110)	(n = 167)	(n = 50)	
**All abnormal nerve US**	53% (30)	45% (50)	**48% (80)**	16% (8)	0.000056
*Wedge-sickle sign*	47% (27)	39% (43)	**42% (70)**	8% (4)	
Fibromuscular	24% (14)	22% (24)	**23% (38)**	0% (0)	
Bony (1^st^ rib)	23% (13)	17% (19)	**19% (32)**	8% (4)	
*Medial insertion of middle scalene*	11% (6)	12% (20)	**16% (26)**	10% (5)	
*Scalenus minimus*	7% (4)	1% (1)	**3% (5)**	0	
**Abnormal vascular US**	93% (53)	81% (89)	**85% (142)**	14% (7)	< 0.00001
**Nerve *and / or* vascular US abnormal**	98% (56)	93% (102)	**95% (158)**	26% (13)	< 0.00001
**Nerve *and* vascular US abnormal**	46% (26)	30% (33)	**35% (59)**	4% (2)	0.000015
**Abnormal angiography**	84% (48)	N/A	N/A	N/A	

Percentage values indicate % of subjects who show abnormality, followed by the absolute number of subjects in parenthesis (n); US: ultrasound; N/A: not applicable; P values indicate chi-square statistics calculated with the pooled patient group (sites A+ B) and the control group

**Table 3:** Sensitivity, specificity, positive predictive values and negative predictive values of ultrasonographic findings in TOS.

	Sensitivity	Specificity	PPN	NPV
**Nerve US (pooled)**	**48%**	**84%**	**91%**	**33%**
*Site A*	53%		79%	61%
*Site B*	45%		88%	41%
**Vascular US (pooled)**	**85%**	**86%**	**95%**	**63%**
*Site A*	93%		88%	91%
*Site B*	81%		93%	67%
**Combined nerve and vascular US (pooled)**	**94%**	**74%**	**92%**	**80%**
*Site A*	98%		81%	97%
*Site B*	93%		89%	82%

Specificity values were the same on both sites, as the same control group was used.

US: ultrasound; PPN: positive predictive value; NPV: negative predictive value

**Fig 5:**
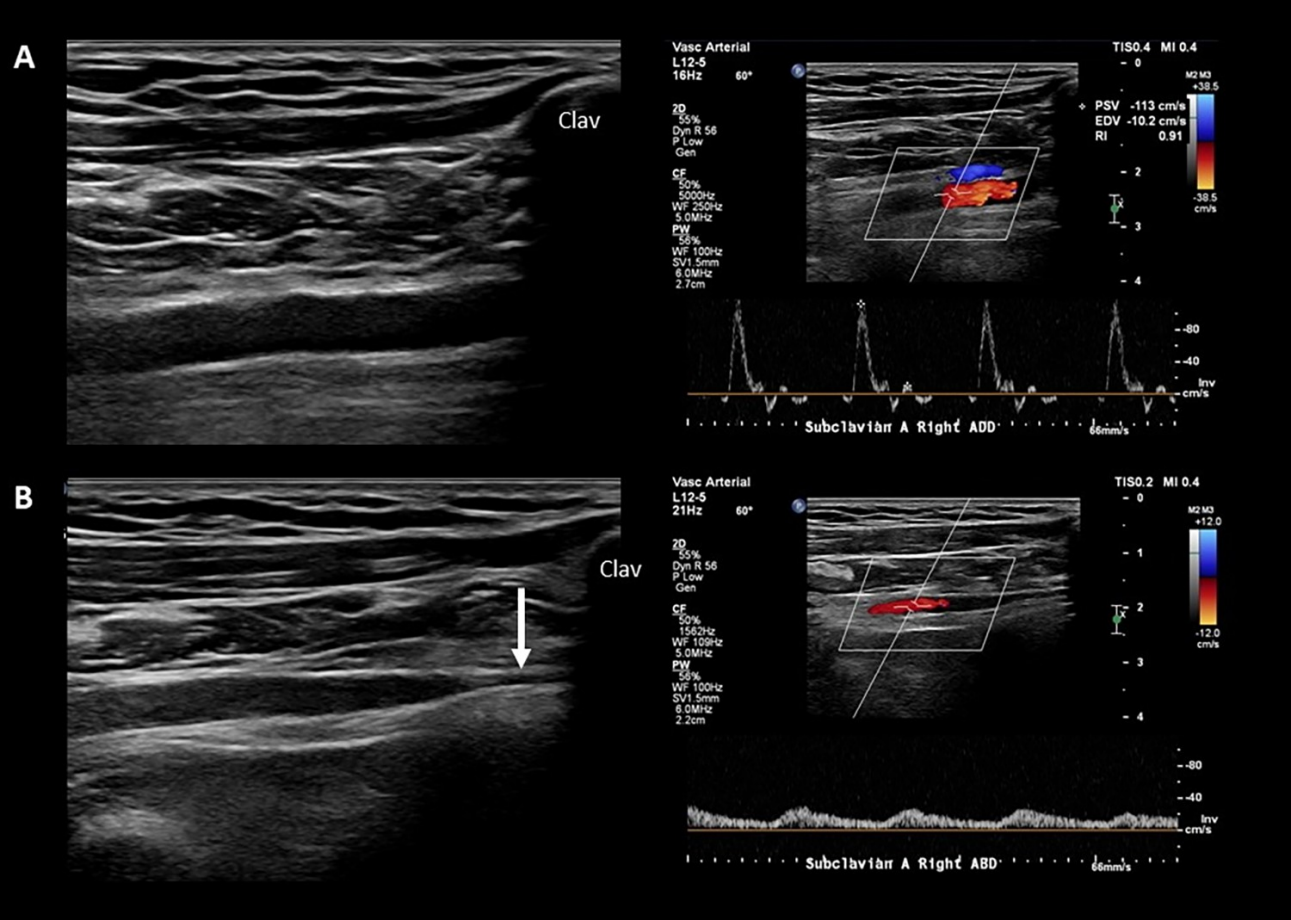
Arterial measurements in TOS. Vascular ultrasound (B mode and PW mode) of the right subclavian artery of a patient with arterial (supra- + costoclavicular) TOS. **A** shows the artery in the immediate infraclavicular region in neutral arm position, and **B** during arm abduction. In the neutral position, normal triphasic flow is seen. Note that during arm abduction the artery becomes severely narrowed (arrow, ‘funnel sign’) as it leaves the costoclavicular space, associated with a tardus-parvus type postocclusion blood flow, loss of pulse, and severe diffuse pain and numbness of the arm. Clav: clavicula.

The proportions of the types of hemodynamic abnormality (not shown in Table 2) were 76/142, 56/142, and 10/142 for occlusion, significant stenosis, and poststenotic flow, respectively. The hemodynamic abnormality occurring during the AER manoeuvre was always associated with exacerbation of the clinical signs, and less consistently with narrowing of the lumen of the subclavian artery immediately distal to the clavicle followed by a poststenotic dilation, resembling a funnel (*Figs 4* and *5*). The ‘funnel sign’ without hemodynamic consequences was also commonly observed in the control group, but the degree of narrowing and its prevalence were lower. This parameter was not used for the analysis. Aneurysm of the subclavian artery was not seen in this cohort.

### Angiography

**Fig 6:**
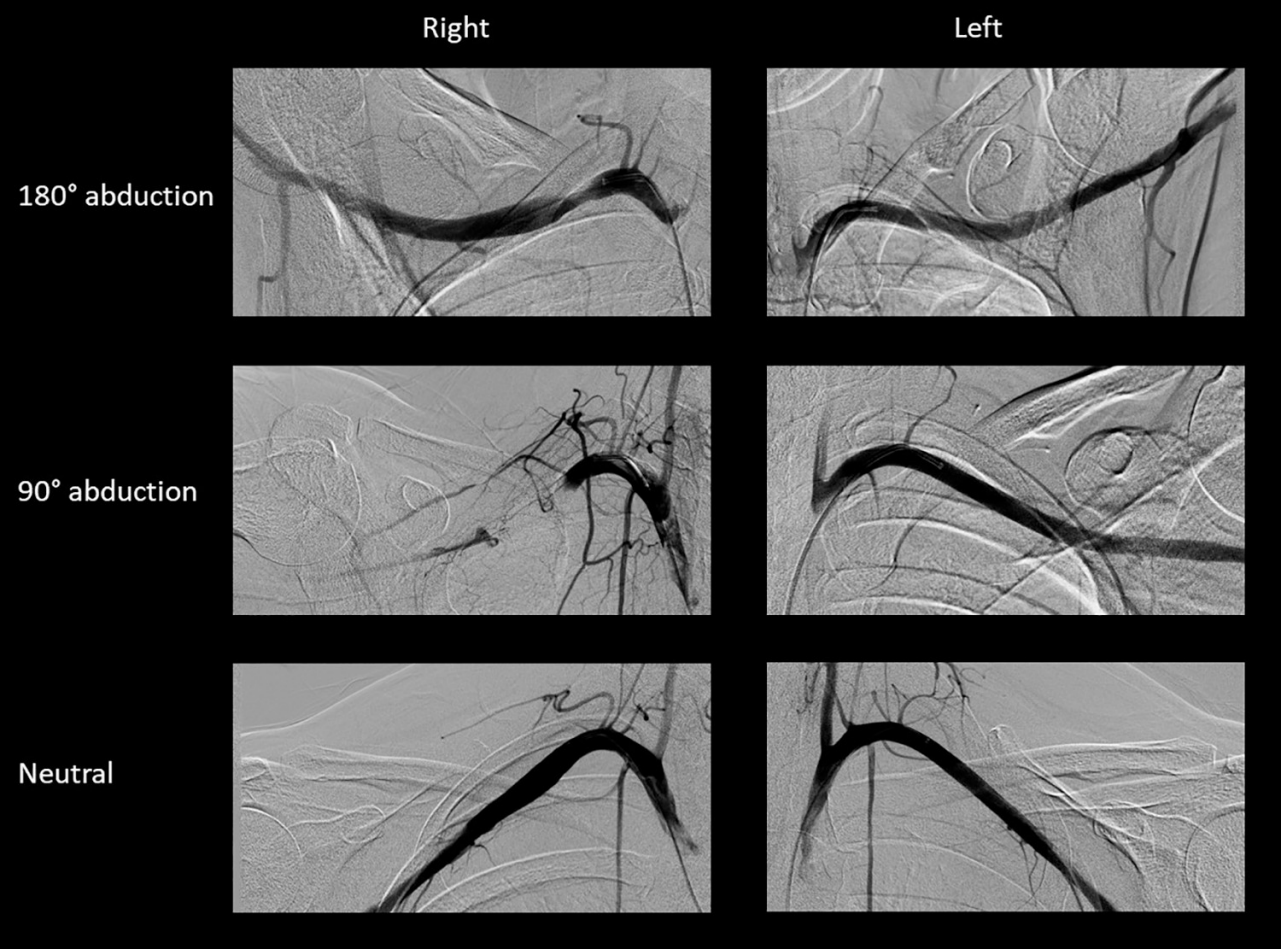
Angiography of the subclavian artery in TOS. Angiography of the subclavian artery of a patient with arterial TOS on the right side. Note the complete occlusion of the right artery when the arm is in 90° abduction, and the stenosis with poststenotic dilation when in 180° abduction.

Angiography was performed on site A and correlated with the results of vascular ultrasound. Compression of the subclavian artery during the AER position was observed in 84% of patients (48/57) (*Fig 6*). Out of the 9 patients in whom the angiography appeared normal, 6 showed abnormal and 3 normal ultrasound results. On the other hand, in only one patient with abnormal angiography was ultrasound measurement normal.

### TOS categories

**Fig 7:**
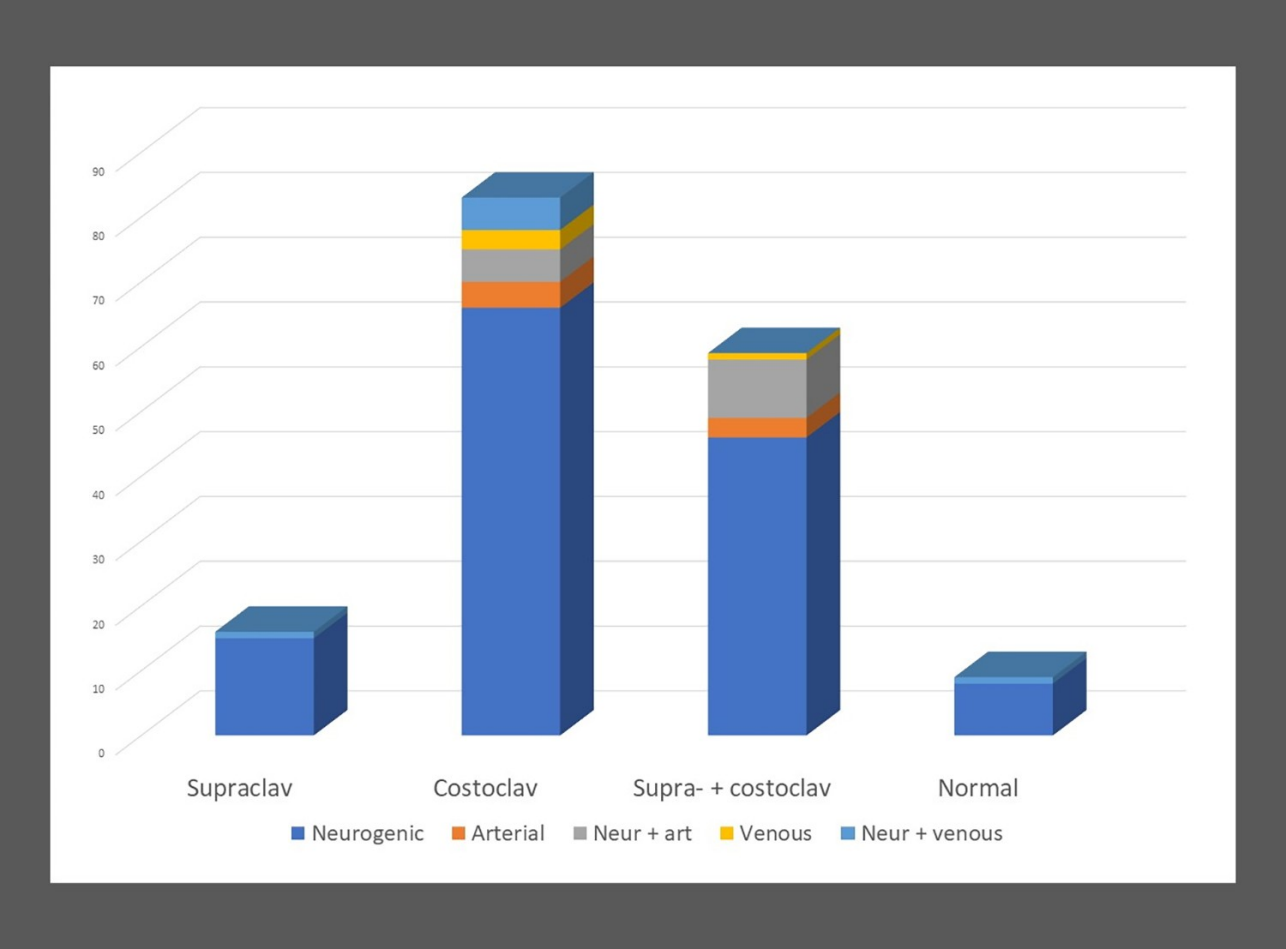
Type of symptoms and site of compression in TOS. Note that the most common *site* of compression was the costoclavicular space, and the most common *type* was neurogenic TOS. The Y axis denotes the number of patients.

The *site* (level) of compression in TOS was determined based on the ultrasound abnormality. Thus, the categories of supraclavicular (abnormal nerve ultrasound), costoclavicular (abnormal vascular ultrasound) or supra- + costoclavicular TOS (abnormal nerve and vascular ultrasound) were set up and their frequency determined. *Fig 7* (pooled data) shows that the most common site of compression was the costoclavicular space, followed by the combined compression at both the supra- and costoclavicular level.

The *type* of TOS was determined based on the clinical symptoms [8] as a gold standard. Pain-numbness and/or neurological deficit in lower trunk distribution, and spontaneous pain in the shoulder, arm or periscapular region were considered as symptoms of neurogenic TOS. Diffuse pain, numbness, pallor, and coldness of the arm, particularly when lifting or exercising the arm indicated arterial TOS. Earlier or ongoing thrombosis of the subclavian vein was the criterion for venous TOS. In addition to the traditional neurogenic, arterial and venous TOS categories, categories with the combination of neurogenic and vascular TOS were also established. The sites and the types of TOS were correlated. Neurogenic TOS (136/167, 81%) was by far the most frequent type of TOS at all sites, followed by combined neurogenic and arterial TOS (14/167, 8%). It is seen in *Fig 7* that the relative proportion of pure neurogenic TOS was the highest in supraclavicular TOS (93%) and in patients with normal ultrasound (100%). On the other hand, 66/133 (50%) neurogenic TOS patients showed only costoclavicular (vascular) abnormality. No significant correlation was found between the site and type of TOS (p = 0.520125).

## Discussion

In the assessment of patients with symptoms suggestive of TOS, high resolution ultrasound of the supraclavicular brachial plexus may visualize congenital fibromuscular / bony anomalies in the scalenic region. The brachial plexus is not accessible for ultrasound within the costoclavicular space, but with the help of dynamic vascular ultrasound of the subclavian vessels in the immediate infraclavicular region, compression within the costoclavicular space can also be ascertained. Although the latter method only measures the vascular component, it may be assumed that the neurovascular bundle comes under compression as a whole. As a follow-up to our previous study investigating supraclavicular ultrasonographic anomalies associated with TOS [7], we have presently undertaken to analyse a larger cohort of patients with neurogenic and/or vascular TOS symptoms combining supraclavicular nerve ultrasound of the brachial plexus with dynamic vascular ultrasound of the infraclavicular subclavian artery. Using clinical symptoms as a gold standard, the results of our study show that the diagnostic sensitivity of the combined nerve and vascular ultrasound is remarkably high (94%) in TOS, and higher than that of each separately.

Concerning supraclavicular anomalies, Roos was the first to divert attention away from cervical ribs to anomalous fibromuscular bands with or without a cervical rib as the real culprit of neurogenic TOS [13–15]. Roos described altogether 14 fibromuscular anomalies, such as fibromuscular bands extending between a rudimentary cervical rib or elongated transverse process of the 7^th^ cervical vertebra and the 1^st^ rib (Types 1 and 2, respectively), or the hard, fibrous medial edge of the middle scalene muscle (Type 3). These anomalies most often cause compression of the lower trunk, which is located in the deepest and narrowest part of the supraclavicular fossa, in an angle formed by the 1^st^ rib (deep), the subclavian artery (medial) and the middle scalene muscle (lateral). We have previously shown that the compression of the lower trunk can be visualized by high resolution ultrasound and described a distinctive ultrasonographic sign called ‘wedge-sickle sign’ [7] (see the caption of *Fig 1* for description). In the present study, the ‘wedge-sickle sign’ was observed in 42% of patients with TOS symptoms, which is of lower prevalence than in our previous study, probably related to the considerably higher sample number and the inclusion of patients with only vascular symptoms. Presently, we have further classified the ‘wedge-sickle sign’ into the fibromuscular and the bony types. The fibromuscular type (*Figs 1* and *4*) indicates compression of the lower trunk by the medial edge of the middle scalene muscle or a fibrous band along the muscle (Roos bands), whereas the bony type is caused by the medial edge of the neck of first rib (*Fig 2*), typically in patients where the first rib is in an elevated, more superficial position. The bony ‘wedge-sickle sign’ was occasionally observed in control subjects as well, but the fibromuscular ‘wedge-sickle sign’ never, rendering it the most specific ultrasonographic marker of the neurogenic component of TOS. Another characteristic supraclavicular finding seen in TOS patients was the broadened insertion of the middle scalene muscle on the 1^st^ rib, extending more medial than usual, thereby narrowing the scalene triangle and contributing to the compression of the lower trunk (*Figs 1* and *3A*). It is thus not surprising that in 16 patients the ‘wedge-sickle sign’ was observed in combination with the medial insertion of the middle scalene muscle. Although fibromuscular structures compressing the lower trunk have also been identified in dedicated MRI studies [16–18], the ease and accessibility of high resolution ultrasound make it the method of choice, in our opinion, for the assessment of the brachial plexus in patients with symptoms suggesting TOS.

The supraclavicular findings, specifically the ‘wedge-sickle sign’, do not by far encompass the whole population of TOS patients, notably those with neurogenic symptoms and a normal supraclavicular ultrasound or those with only vascular symptoms. The neurovascular bundle may also become compressed further distal, particularly in the costoclavicular space. As opposed to the static nature of the supraclavicular anomalies detailed above, costoclavicular compression is typically dynamic in nature, occurring when the arm is abducted or engaged in some activity, a well-known feature of TOS. Imaging studies have demonstrated that during hyperabduction and external rotation of the arm (AER) the clavicula moves in the posterior direction, causing significant narrowing of the costoclavicular interval, but not the scalene triangle [19–22]. Although this space is inaccessible to ultrasound, changes in the blood flow of the subclavian artery and vein in the immediate infraclavicular region, assessed by Duplex ultrasound, may indicate impingement proximally in the costoclavicular interval [23, 24]. The diagnostic utility of this method has been questioned by the relatively common occurrence of dynamic compression of the subclavian artery in asymptomatic individuals, ranging from 20–30% in the literature [23, 25, 26], and being 14% in our study. However, in TOS patients the vascular changes are always accompanied by the exacerbation of the patients’ usual clinical symptoms. In the present study, the sensitivity of vascular ultrasound was 93% and 81% on Sites A and B, respectively, the marginally significant difference probably related to referral bias on the two sites. The hemodynamic changes were typically associated with the flattening of the artery, termed as the ‘funnel sign’ (*Figs 4*, *5*). On Site A, angiography was also performed, which had a slightly lower sensitivity, but otherwise showed a good correlation with ultrasonographic findings. The pooled sensitivity of vascular ultrasound was 85%, as opposed to 48% for nerve ultrasound, suggesting that costoclavicular compression is more common than supraclavicular anomaly in TOS. As none of the patients had trauma or other acquired pathology in the region, and only one patient had a clear congenital 1^st^ rib anomaly (a hypoplastic, elevated 1^st^ rib articulating with the 2^nd^ rib), the narrowing of the costoclavicular space in most patients with idiopathic TOS is probably related to subtle bony anomalies, e.g. a J-shaped, elevated 1^st^ rib [27–29], muscle hypertrophy or external factors, such as bad posture, occupational or recreational activities, alone or in combination.

Regarding the *type* of TOS, based on clinical symptoms as a gold standard, neurogenic TOS was by far the most common type, in accordance with the literature. However, we recommend also to determine the *site(s)* of compression (i.e. the cause of TOS), as it has obvious therapeutic consequences. Based on the abnormalities of nerve and vascular ultrasound in our study, the most common site of compression was the costoclavicular space, followed by the combined compression at both the supra- and costoclavicular level (*Fig 7*). Our results highlight that the cause of TOS is often multilevel. Furthermore, vascular ultrasound was the only abnormality in 50% of neurogenic TOS patients, underscoring the role of dynamic vascular ultrasound in identifying the costoclavicular space as the site of compression of the neurovascular bundle even in patients with neurogenic TOS. As the brachial plexus cannot be assessed in the costoclavicular space by ultrasound, dynamic arterial compression may be used as a marker for this site. The coexistence of arterial compression in patients with neurogenic thoracic outlet syndrome has been addressed previously in a limited number of studies, using Duplex ultrasound [24, 30, 31] or other imaging methods [32, 33]. They also underline, as observed in our study, that arterial involvement does not necessarily mean the occurrence of thrombosis or aneurysm of the subclavian artery, which is the prevailing strict definition of arterial TOS [3].

It should be mentioned here that an on-going controversy exists in the literature regarding both the prevalence and the categories of TOS. Traditionally, in the neurological literature neurogenic TOS is diagnosed only when objective signs of lower trunk lesion are present (labelled as ‘true neurogenic TOS’) [2, 34–36], which is considered to be a very rare entity with an estimated prevalence of 1 per one million [36]. Patients with only subjective symptoms (e.g. pain and paraesthesia in lower trunk distribution) without neurologic / electrophysiological deficit were labelled as ‘disputed TOS’ [6]. On the other hand, the surgical literature includes these patients in the category of neurogenic TOS, the symptoms being attributed to the irritation of the brachial plexus, and considers it a common condition with a prevalence ranging from 3 to 80 per 1,000 [37, 38]. Our study shows that objective sonographic signs are present in the majority of patients labelled as ‘disputed or nonspecific TOS’, and we discourage the use of this category. Moreover, these patients appear to constitute the bulk of TOS patients, as 81% of our patients had no neurological deficit and none had signs of arterial disease. In summary, the typical TOS patient appears to be a young female with neurogenic TOS symptoms, but no neurological deficit, and with compression at the costoclavicular space.

Limitations of our study include the retrospective nature of the study, as well as the lack of analysis of venous blood flow and the retropectoral space. Although venous measurements were performed in most patients, but not as systematically as arterial measurements, and thus they were not included in the analysis. In one study postural venous flow anomalies were found in 60% of normal volunteers [25], rendering it perhaps a less appropriate candidate as a marker for costoclavicular compression in TOS.

## Conclusion

Our study demonstrates the high diagnostic sensitivity and reliability of combined nerve and vascular ultrasound in patients with clinical symptoms suggestive of TOS, being higher than that of nerve or vascular ultrasound alone. Moreover, combined nerve and vascular ultrasound can identify the site of compression in TOS, which has obvious therapeutic consequences, and should be determined in addition to the type of TOS symptoms. Sonography of the supraclavicular brachial plexus may visualize musculoskeletal anomalies affecting the brachial plexus in the scalenic region, whereas the dynamic hemodynamic compromise of the infraclavicular subclavian artery may be regarded as a marker for the costoclavicular impingement of the neurovascular bundle. We found that the costoclavicular space is the most frequent site of compression, but the compression is not uncommonly multi-level.

## Supporting information

S1 Video The fibromuscular ‘wedge-sickle sign’ combined with the anomalous insertion of the middle scalene muscle. Caudocranial axial scanning of the right brachial plexus of the patient shown in Fig 1. Note the medial insertion of the middle scalene muscle on the first rib. More cranially, the wedge-shaped hyperechoic structure at the medial edge of the muscle indents the hypoechoic lower trunk, seen on the lateral side of the subclavian artery. Further cranially, this fibromuscular structure ends at the hyperechoic bony anterior tip of a rudimentary cervical rib (Type 1 Roos ligament).
(MP4)
